# Genome-Wide Identification and Expression Profiling of the PYL Gene Family in Watermelon Under Abiotic Stresses

**DOI:** 10.3390/genes17040426

**Published:** 2026-04-04

**Authors:** Guangpu Lan, Yidong Guo, Jun Hu, Jincan Huang, Ziye Pan, Yingda Chen, Xian Zhang, Zhongyuan Wang, Yongchao Yang, Chunhua Wei

**Affiliations:** 1 Hainan Institute of Northwest A&F University, Sanya 572024, China; guangpulan@nwafu.edu.cn (G.L.); zydx@nwafu.edu.cn (Z.W.); 2 State Key Laboratory of Crop Stress Biology in Arid Areas, College of Horticulture, Northwest A&F University, Yangling 121100, China; 3 College of Biological and Agricultural Sciences, Honghe University, Mengzi 661199, China

**Keywords:** watermelon, *PYL* gene family, ABA receptors, *cis*-acting elements, abiotic stresses

## Abstract

**Background:** PYR/PYL/RCAR proteins are core abscisic acid (ABA) receptors that play essential roles in ABA signal transduction, plant growth and development, and abiotic stress responses. However, the *PYL* gene family in watermelon (*Citrullus lanatus*) has not been systematically characterized, limiting our understanding of ABA-mediated stress adaptation in this economically important crop. **Methods:** A genome-wide analysis was performed to identify *ClPYL* genes in watermelon using a hidden Markov model search. Phylogenetic relationships were reconstructed using the maximum likelihood method. Segmental duplication events were analyzed using synteny analysis. Conserved motifs, gene structures, and promoter *cis*-acting elements were characterized using MEME and PlantCARE. Expression profiles under drought, salt, and cold stresses were examined by quantitative real-time PCR (qRT-PCR) with three biological replicates. **Results:** In this study, 15 *ClPYL* genes were identified in watermelon through genome-wide analysis. Phylogenetic reconstruction classified these genes into four subfamilies, with subfamily II being exclusively present in cucurbits—a lineage-specific feature not observed in Arabidopsis. Synteny analysis revealed eight segmental duplication events involving members of subfamilies I, III, and IV, while subfamily II members were not associated with these duplications. Members within the same subfamily share similar exon-intron structures and conserved motifs. Promoter analysis revealed that *ClPYL* genes are enriched with various *cis*-acting elements associated with hormone signaling and abiotic stress responses. Expression profiling demonstrated that *ClPYL* genes exhibit diverse and dynamic expression patterns under drought, high-salinity, and cold stresses. Notably, genes such as *ClPYL5* under drought, *ClPYL02* under salt, and *ClPYL15* under cold stress displayed persistent stress-responsive expression. **Conclusions:** These findings reveal the evolutionary conservation and diversification of the *PYL* family in watermelon and provide a set of candidate genes for functional studies aimed at dissecting ABA-mediated stress adaptation. This work establishes a genomic framework for developing stress-resilient watermelon varieties through molecular breeding.

## 1. Introduction

Abscisic acid (ABA) was initially discovered in the 1960s in some woody perennials and cotton, where it was found to significantly induce bud dormancy in the woody plants and promote abscission of both fruits and leaves in cotton (*Gossypium hirsutum*) [1]. ABA is recognized as a key phytohormone in plants, plays a critical role in seed dormancy, growth inhibition, and leaf senescence, as well as serving as an integral component of plant defense mechanisms [2,3]. Notably, under adverse conditions such as drought, high salinity, and low temperature, ABA levels rapidly increase, leading to growth inhibition and prioritized resource allocation to cope with stress [4,5,6].

Under stress conditions, elevated ABA levels trigger a signaling cascade that enables plants to adapt and survive, making ABA signaling a critical pathway for stress tolerance [7]. ABA receptor PYR/PYL/RCAR belongs to the START domain superfamily, featuring a conserved hydrophobic ligand-binding pocket [8,9]. Upon ABA binding, PYL receptors undergo conformational changes that enable their interaction with type 2C protein phosphatases (PP2Cs), thereby relieving the inhibition of SNF1-related protein kinases (SnRK2s) and activating downstream stress-responsive transcription factors and physiological responses [8,10,11,12]. Structural analyses have revealed that PYL receptors exhibit diverse oligomeric states which contribute to their functional versatility in regulating plant growth and stress adaptation [13,14,15,16]. Due to their pivotal position in the ABA signaling cascade, *PYL* genes have emerged as critical targets for understanding and improving plant stress tolerance.

This 14-member gene family (*Pyr1* and *Pyl1* to *Pyl13*) has been independently identified, and mutant lines reveal ABA insensitivity in *Arabidopsis* [9]. The *PYL* gene family has been identified in multiple species, with wheat (*Triticum aestivum*) containing 38 members [17], cotton (*Gossypium hirsutum*) 27 members [18], tomato (*Solanum lycopersicum*) 22 members [19], luffa (*Luffa cylindrica*) 14 members [20], cucumber (*Cucumis sativus*) 14 members [21], and melon (*Cucumis melo*) 13 members [22].

Watermelon (*Citrullus lanatus* L.) is an economically important crop cultivated worldwide, yet its production is threatened by climate change-induced abiotic stresses such as drought, salinity, and extreme temperatures. While ABA receptor *PYL* genes have been extensively studied in numerous plant species, no systematic investigation exists for the watermelon *PYL* family, including its evolutionary dynamics, structural features, and expression patterns under stress conditions. This knowledge gap limits our understanding of how watermelon perceives and transduces stress signals through the ABA pathway and hinders the exploitation of PYL genes for crop improvement. Here, we identified 15 *ClPYL* genes and analyzed their phylogenetic relationships, chromosomal distributions, gene structures, conserved motifs, and promoter *cis*-elements. Expression profiling of *ClPYLs* under various abiotic stresses was further performed. Our results reveal that *ClPYL* genes exhibit subfamily-specific structural features and stress-responsive expression patterns. This work provides a foundational resource for elucidating the molecular functions of *PYL* genes in watermelon stress responses and offers valuable targets for breeding stress-tolerant varieties.

## 2. Materials and Methods

### 2.1. Identification and Chromosomal Distribution of ClPYL Genes

To identify the *PYL* gene family in watermelon, the complete protein sequence file of the *Citrullus lanatus* line “97103” (V2) was downloaded from the Cucurbit Genomics Database (CuGenDB; http://cucurbitgenomics.org/, accessed on 15 October 2024). The hidden Markov model (HMM) profile corresponding to the PYL domain (Pfam accession: PF10604) was obtained from the Pfam database (http://pfam.xfam.org/, accessed on 15 October 2024). A genome-wide search was then performed using the hmmsearch program (v3.3.2) against the watermelon protein dataset with an E-value threshold of 1× 10^−5^. All candidate sequences were verified by searching against the Pfam and NCBI Conserved Domain Database (CDD) to confirm the presence of the complete PYL domain. Redundant sequences were manually removed based on gene IDs and chromosomal locations. Finally, the chromosomal locations of the identified *ClPYL* genes were determined and visualized using the software TBtools (v2.206).

### 2.2. Analysis of Gene Structure, Syntenic, and Phylogenetic Evolution of ClPYL Genes

To investigate the gene structure of the *ClPYLs*, conserved motifs were predicted using the protein sequences of *ClPYLs* via the online tool MEME (https://meme-suite.org/, accessed on 30 October 2024). The analysis was configured to identify 10 motifs with widths ranging from 6 to 50 amino acids [23]. Gene structure information (exon-intron organization) was extracted and visualized using the software TBtools.

The protein sequences of previously reported *PYL* genes from Arabidopsis (14 AtPYLs) and melon (13 CmPYLs) were retrieved from the Arabidopsis Information Resource (TAIR; https://www.arabidopsis.org/) (accessed on 30 October 2024) and the CuGenDB database accessed on 30 October 2024, respectively. Protein sequences were used for phylogenetic reconstruction due to their higher evolutionary conservation, lower susceptibility to saturation and homoplasy, and greater phylogenetic informativeness compared to nucleotide sequences, particularly for distantly related species [24]. A multiple sequence alignment was performed using ClustalW default parameters, followed by manual trimming to remove poorly aligned regions and gap-rich positions using MEGA 7.0 software [25]. A phylogenetic tree was constructed using the maximum likelihood method in MEGA with 1000 bootstrap values to assess the robustness of the tree topology.

To investigate syntenic relationships within the *ClPYL* gene family, genome-wide collinearity analysis was performed using MCScanX [26]. The input files were prepared based on BlastP alignment results with an E-value threshold of 1 × 10^−5^ and the corresponding GFF annotation file. Segmental duplication events were identified by evaluating syntenic blocks containing at least five collinear gene pairs. The resulting collinear relationships were visualized using CIRCOS (http://circos.ca/, accessed on 30 October 2024).

### 2.3. Prediction of Cis-Acting Elements in ClPYL Promoters

To identify potential *cis*-acting elements within the promoters of *ClPYL* genes, the 2.0 Kb genomic sequences upstream of the transcription start site of each *ClPYL* were extracted. Promoter analysis was performed using the online database PlantCARE (https://bioinformatics.psb.ugent.be/webtools/plantcare/html/) (accessed on 30 October 2024) to predict putative *cis*-regulatory elements [27]. Following the prediction, the distribution of selected elements was visualized using TBtools.

### 2.4. Expression Profiling of ClPYLs Under Drought, Cold, and Salt Stresses

The experiment was conducted in a sunlight greenhouse using the watermelon male-fertile inbred line ‘YL’ as the plant material. This line is widely used in genetic and breeding studies due to its stable agronomic traits and suitability for stress physiology research [28,29]. Seeds were first treated with hot water (55–65 °C) for 4 h, then germinated in a dark incubator at 28 °C with high humidity for 30–48 h until radicle emergence. Germinated seeds were sown in nutrient pots (8 × 7 × 7 cm ^3^) and grown on a culture shelf under an 18 h/6 h light/dark cycle with temperatures of 30 °C (light) and 16 °C (dark). Plants were watered routinely until they reached the four-leaf stage.

In total, 90 uniform seedlings per treatment were selected. Drought stress was imposed by withholding water for 8 days, with sampling at 0, 2, 4, 6, and 8 days. To ensure uniform and consistent drought conditions, all pots were filled with equal amounts of homogenized substrate, and the substrate water content was maintained at approximately 75% of field capacity before stress initiation. Drought severity was assessed based on the duration of water withholding and the appearance of visible wilting symptoms at each sampling time point. Salt stress was applied by irrigating each seedling with 100 mL of 300 mM NaCl solution, and samples were collected at 0, 8, 18, 30, 42, and 54 h after treatment. The concentration of 300 mM NaCl was selected based on its widespread use in salt stress studies in watermelon [30,31]. For cold stress, seedlings were transferred to a 4 °C chamber and sampled at 0, 6, 12, 24, and 48 h. Each treatment included three biological replicates. The second true leaf from the growing point was collected per seedling. Leaves from the same replicate were pooled, immediately frozen in liquid nitrogen, and stored at –80 °C for subsequent RNA extraction.

Total RNA was extracted using the RNA Simple Total RNA Kit (TIANGEN, Beijing, China). cDNA synthesis was performed with the FastKing RT Kit (TIANGEN, Beijing, China), and quantitative real-time PCR (qRT-PCR) was carried out using SYBR Green Master Mix (Vazyme Nanjing, China). Three biological replicates were performed for each time point, with three technical replicates per biological replicate. The watermelon Actin gene (Cla97C02G026960) was used as an internal control, and relative expression levels were calculated using the 2^−ΔΔCt^ method [32]. For heatmap visualization, the relative expression values were transformed to log_2_ scale. Genes were considered to be differentially expressed if they met the criteria of *p* < 0.05 or *p* < 0.01, as determined by Student’s *t*-test comparing each time point to the respective control in SPSS 19.0 software. All primers used in this study are listed in Table 1.

## 3. Result

### 3.1. Identification and Chromosomal Distribution of ClPYL Genes in Watermelon

**Figure 1:**
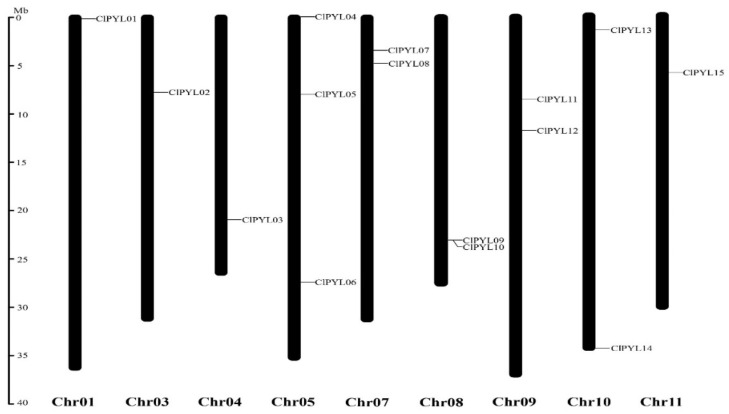
Chromosome distribution of *ClPYLs* in watermelon. The relative positions of *ClPYLs* are marked on the chromosomes. According to their physical location on the chromosomes, the *PYL* genes of watermelon are named from *ClPYL01* to *ClPYL15*, which corresponds to the gene’s ID.

Using a Hidden Markov Model (HMM), we identified 15 candidate *ClPYL* genes in the watermelon genome. Chromosomal position-based nomenclature designated these as *ClPYL01*~*ClPYL15*, with corresponding gene IDs and locations detailed (Table 2). The encoded proteins range from 162 to 233 amino acids in length, corresponding to molecular masses of 17.7~25.4 kDa. While the majority (13 of 15) have an acidic isoelectric point (pI < 7), the remaining two proteins exhibit a basic pI (>7) (Table 2). Chromosomal distribution analysis revealed that *ClPYL* genes are localized to nine chromosomes. Chromosomes 1, 3, 4, and 11 each contain a single *ClPYL* gene, while chromosomes 7, 8, 9, and 10 each carry two. Notably, chromosome 5 harbors the highest number, with three *ClPYL* genes (Figure 1). Thus, the 15 *ClPYL* genes are unevenly distributed across nine watermelon chromosomes.

### 3.2. Phylogenetic and Synteny Analysis of ClPYLs

**Figure 2:**
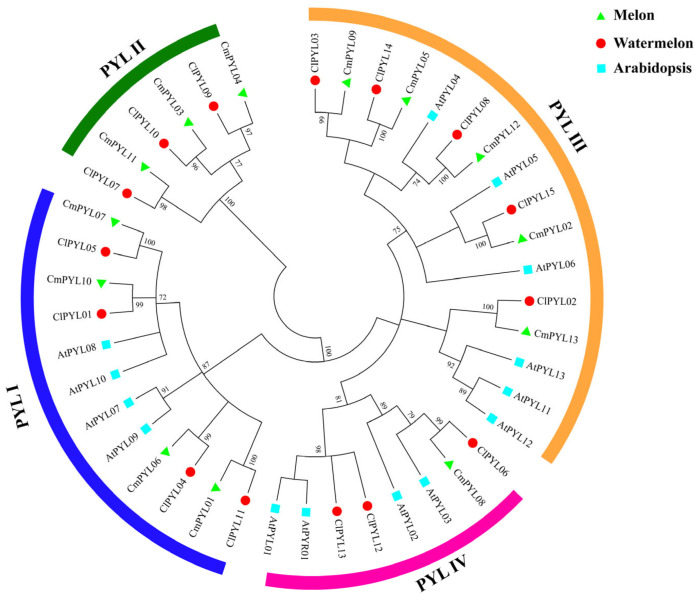
Phylogenetic tree of the *PYL* genes in watermelon, melon, and Arabidopsis by the maximum likelihood (ML) method using MEGA software. The *PYL* members were categorized into four clades and labeled as I, II, III, and IV. Cl: Watermelon (red circle), Cm: Melon (green triangle), At: Arabidopsis thaliana (blue square).

To elucidate the evolutionary relationships of *ClPYLs* with other species, we constructed a phylogenetic tree using protein sequences of the 15 *ClPYLs*, together with 13 *CmPYLs* from melon, and 14 *AtPYLs* from Arabidopsis (Figure 2). The PYL family segregates into four monophyletic clades (I–IV) for high bootstrap support (≥70% for most major branches), where clade III represents the largest subgroup and clade II the smallest. The clade I consisted of four *ClPYLs*, four *CmPYLs*, and four *AtPYLs*. The clade II consisted of three *ClPYLs* and three *CmPYLs*. The clade III consisted of five *ClPYLs*, five *CmPYLs*, and six *AtPYLs*. The clade IV consisted of three *ClPYLs*, one *CmPYL*, and four *AtPYLs*. A particularly noteworthy finding is that clade II contains exclusively cucurbit PYLs, comprising three orthologous pairs: *ClPYL10*-*CmPYL3*, *ClPYL09*-*CmPYL4*, and *ClPYL07*-*CmPYL11* with no representatives from Arabidopsis. This cucurbit-specific subfamily (II) represents a lineage-restricted subfamily that distinguishes it from the three subfamilies (I, III, and IV) shared more broadly across angiosperms. Phylogenetic reconstruction reveals stronger conservation between watermelon and melon of *PYLs* than with Arabidopsis. Collectively, these results reveal the presence of a cucurbit-specific *PYL* subfamily alongside three subfamilies shared with Arabidopsis, highlighting both conserved and lineage-specific phylogenetic patterns of the *PYL* gene family.

**Figure 3:**
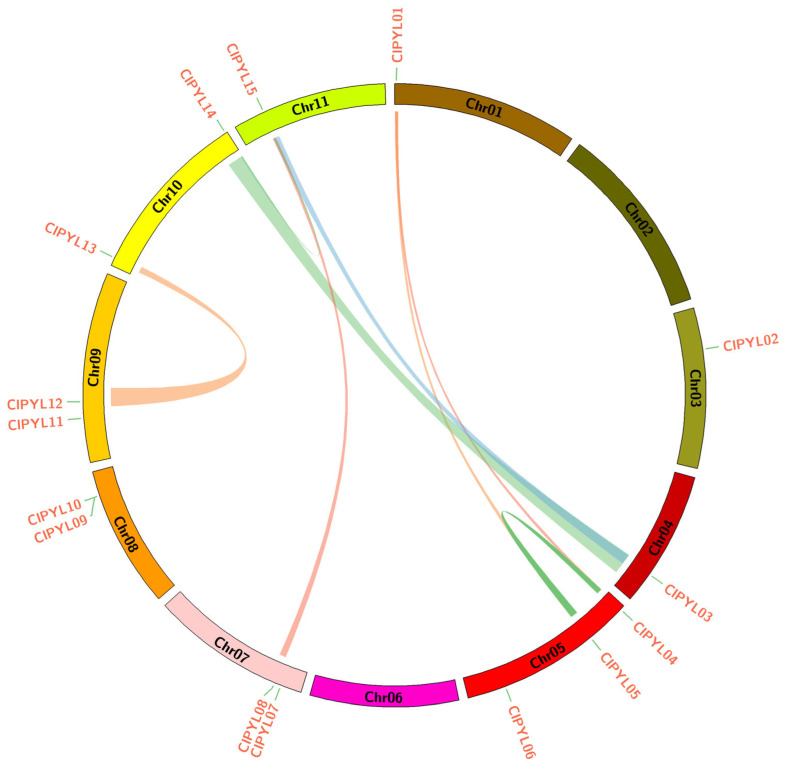
Segmental duplication events of *CIPYL* genes in watermelon. The Circos plot visualizes the genomic localization of 15 *CIPYL* genes across 11 chromosomes (colored blocks). Segmental duplication pairs are represented by connecting lines between collinear chromosomal regions, revealing 8 pairs of duplication events.

Synteny analysis using MCScanX revealed that the 15 *ClPYL* genes are unevenly distributed across 11 chromosomes (Chr01–Chr11) in watermelon, with Chr05 harboring the largest number (three genes). A total of eight pairs of segmental duplication events were identified, and 9 *ClPYL* genes from subfamilies I, Ⅲ, and IV were located within collinear regions (Figure 3). These duplications occurred both interchromosomally (seven events) and intrachromosomally (one event on Chr05), suggesting that segmental duplication is the primary driver of expansion for these three subfamilies and has contributed substantially to the structural diversification of the *ClPYL* gene family.

### 3.3. Structure and Conserved Domain Analysis of ClPYLs

**Figure 4:**
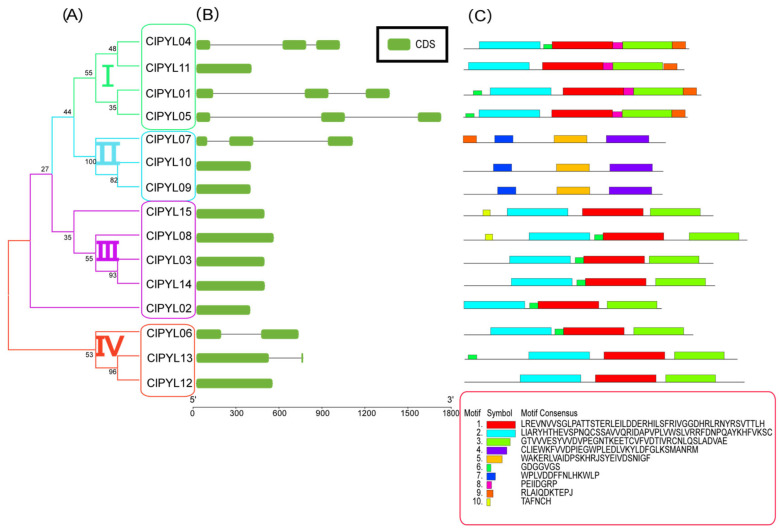
Motifs and gene structure analysis of the *ClPYL* gene family. (**A**) The phylogenetic tree of *ClPYLs* using the neighbor-joining method. (**B**) The *PYL* gene family structure of watermelon. The green squares are CDS. (**C**) Conserved sequence analysis of the *PYL* gene family in watermelon.

Phylogenetic analysis classified the *ClPYLs* into four subfamilies, each containing at least three members (Figure 4A). Gene structure analysis revealed variation in exon number across subfamilies (Figure 4B). Subfamily I members predominantly contain three exons, with the exception of *ClPYL11*, which has only one. In Subfamily II, *ClPYL07* contains three exons, whereas *ClPYL09* and *ClPYL10* each contain only one. All members of subfamily III feature a single exon. Subfamily IV includes *ClPYL06* and *ClPYL13* with two exons and *ClPYL12* with one. To identify the characteristic regions of *ClPYLs*, we employed the online tool MEME and identified ten conserved motifs (Figure 4C). The number of motifs in ClPYL proteins ranges from three to six. Motifs 1~3 were present in all ClPYLs except those in subfamily II, which uniquely contained motifs 4, 5, and 7. Based on Pfam database alignment, motifs 3 and 5 were annotated as part of the START domain, supporting the typical structural architecture of the ClPYL family. Additionally, all members of subfamily I contain motif 8 and motif 9. Except for *ClPYL11*, *ClPYL12*, *ClPYL15*, and subfamily II, all members contain motif 6. Only *ClPYL08* and *ClPYL15* contain the motif 10. Overall, these results reveal that exon-intron organization and conserved motif composition are largely consistent within each subfamily but differ among subfamilies.

### 3.4. Analysis of Cis-Acting Elements in the Promoters of ClPYLs

**Figure 5:**
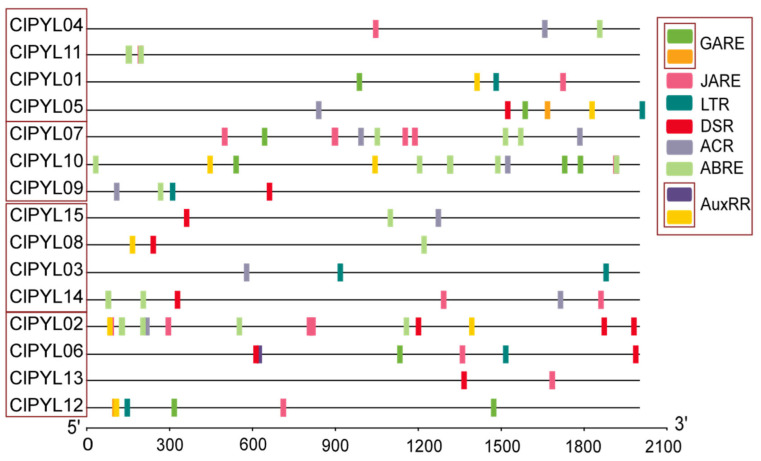
The *cis*-acting element prediction of *ClPYLs*. The promoters of *ClPYLs* include gibberellin-responsive elements (GARE), JA-responsive element (JARE), auxin-responsive element (AuxRR), abscisic acid-responsive element (ABRE), low-temperature-responsive element (LTR), anaerobic response element (ACR), and stress-responsive element (DSR).

To further predict the potential regulatory pathways involving *ClPYLs*, we analyzed the *cis*-acting elements within their 2.0 Kb promoter regions (Figure 5). The results revealed an abundance of *cis*-acting elements, with nine types occurring at high frequency, most of which are responsive to phytohormones and stress. These include the gibberellin-responsive elements (GARE), JA-responsive element (JARE), auxin-responsive element (AuxRR), abscisic acid-responsive element (ABRE), low-temperature-responsive element (LTR), anaerobic response element (ACR), and stress-responsive element (DSR). Notably, the promoters of *ClPYL02*, *ClPYL07*, and *ClPYL10* were identified to contain more than ten *cis*-acting elements each, while those of *ClPYL05*, *ClPYL06*, *ClPYL12*, and *ClPYL14* were found to harbor more than five. This suggests that *ClPYLs* may function in hormone signaling pathways and/or stress responses. Subfamily I promoters were dominated by ABRE and GARE elements, while subfamily II was mainly enriched in ABRE, JARE, and ACR elements. Subfamily III consistently showed high abundance of DSR and ABRE elements, and subfamily IV was characterized by enriched DSR and JARE elements. These results highlight the diversity and subfamily-specific distribution of *cis*-acting elements within the *ClPYL* promoter regions.

### 3.5. Expression Analysis of ClPYLs in Response to Low-Temperature, Salt, and Drought Stresses

**Figure 6:**
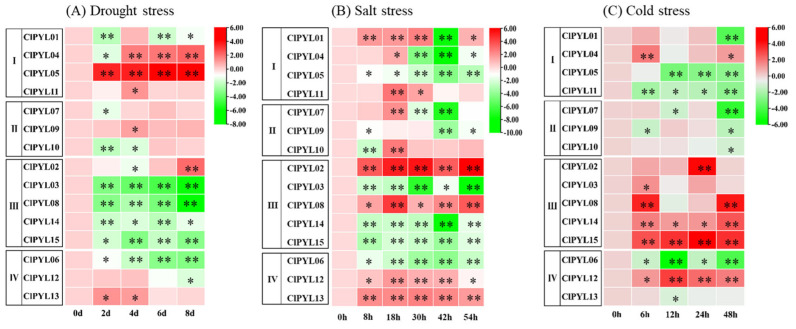
Gene expression heatmap of the *ClPYL* genes in the watermelon leaf under drought (**A**), salt (**B**), and cold stress (**C**). Red: up-regulated. Green: down-regulated. Statistical significance was determined using Student’s *t*-test comparing each time point to the control. Asterisks indicate significant differences: * *p* < 0.05, ** *p* < 0.01.

We profiled the expression of *ClPYL* genes in watermelon seedlings undergoing drought, high-salinity, and low-temperature stresses. Under drought conditions (Figure 6A), the *ClPYL02*, *ClPYL04*, *ClPYL05*, *ClPYL09*, *ClPYL11*, and *ClPYL13* were observed to exhibit increased expression levels. It is noteworthy that *ClPYL05* exhibited induction at all time points. *ClPYL04* showed induction at 4, 6, and 8 days of stress, and *ClPYL02* was markedly induced specifically at day 8. Additionally, *ClPYL09*, *ClPYL11*, and *ClPYL13* were induced at either day 2 or day 4. In contrast, the *ClPYL06* and all members of Subfamily III, except for the *ClPYL02*, were down-regulated under stress.

Exposure to salt stress for 54 h elicited diverse transcriptional responses of the *CIPYL* genes (Figure 6B). The expressions of *ClPYL02*, *ClPYL08*, *ClPYL12*, and *ClPYL13* were significantly up-regulated throughout the stress period. In contrast, *ClPYL01* showed transient induction between 8 and 30 h, followed by a sharp decline at 42 h. An expression up-regulated was observed at 18 h for *ClPYL04*, *ClPYL07*, *ClPYL10*, and *ClPYL11*. Conversely, *ClPYL03*, *ClPYL05*, *ClPYL06*, *ClPYL09*, *ClPYL14*, and *ClPYL15* displayed down-regulation under salt stress.

Under cold stress, distinct expression patterns were observed among *ClPYL* subfamilies (Figure 6C). In subfamily I, all members were down-regulated except for *ClPYL04*, which was up-regulated at 6 h. All members of subfamily II exhibited down-regulation. In subfamily III, all members showed up-regulation, with *ClPYL02* displaying peak expression at 24 h, *ClPYL08* showing the highest expression level at 6 h and 48 h, and *ClPYL15* maintaining consistently high expression throughout the stress period. Within subfamily IV, *ClPYL06* and *ClPYL13* were significantly down-regulated, while the *ClPYL12* was markedly up-regulated. The expression of *ClPYL* genes under diverse abiotic stresses provides candidates for functional validation in plant stress responses.

Notably, the stress-responsive expression patterns of *ClPYL* genes showed some correspondence with their promoter *cis*-element compositions. For example, *ClPYL05*—which exhibited upregulation under drought stress—contains ABRE and DSR elements in its promoter, while *ClPYL12*, which maintained high expression in cold stress, harbors LTR elements. *ClPYL02*, which was upregulated under all three stresses, contains more than ten *cis*-elements in its promoter. These observations suggest a possible association between promoter architecture and stress-responsive expression, although functional validation is required to determine whether these *cis*-elements are directly responsible for the observed transcriptional regulation.

## 4. Discussion

Plants are frequently exposed to various abiotic stresses such as drought, high salinity, and low temperature during their growth and development, which can significantly inhibit growth and cause physiological damage [33]. The ABA signaling pathway is recognized as one of the key mechanisms enabling plants to cope with these stresses [7]. Within this pathway, PYL (PYR/PYL/RCAR) receptors function as core components that perceive ABA and initiate downstream signaling. Therefore, characterizing the *PYL* gene family in plants is of great importance for identifying stress tolerance-related genes.

This study identified a total of 15 *ClPYL* members in the watermelon genome, which are unevenly distributed across nine chromosomes (Figure 1). The number of identified *ClPYL* genes is comparable to that in other economically important cucurbits, such as melon (13 *CmPYLs*) and cucumber (14 *CsPYLs*) [21,22], yet shows variation when compared to more distantly related species like wheat (38 *TaPYLs*) and cotton (27 *GhPYLs*) [17,18]. The difference in *PYL* gene numbers among species is consistent with the broader evolutionary diversity observed in plant gene families. In previous studies, the *PYL* gene family has been classified differently across species. For example, in wheat [17], soybean (*Glycine max*) [34], and sweet potato (*Ipomoea batatas*) [35], *PYL* genes are divided into three subgroups, while in the tea plant [36], they are categorized into five subfamilies, reflecting functional diversity among species. Phylogenetic analysis of the *PYL* genes from watermelon, melon, and *Arabidopsis* classified the *ClPYL* family into four subfamilies (Figure 2). Synteny analysis identified eight segmental duplication events, involving members of subfamilies I and IV, while subfamily II members were not associated with these duplications (Figure 3). While subfamilies I–III comprise members from all three species, subfamily II is exclusively composed of genes from watermelon and melon. This cucurbit-specific clustering highlights the evolutionary distinctiveness of subfamily II genes following the divergence of the cucurbit lineage from Arabidopsis. Notably, these members exhibit several distinctive features. First, they possess unique motif compositions (motifs 4, 5, and 7) that are not present in other subfamilies (Figure 4C). Second, their promoter regions are enriched with multiple hormone-responsive elements, including ABRE (Figure 5). However, their expression patterns under abiotic stresses did not reveal consistent or uniform trends across members; instead, individual genes within subfamily II displayed divergent responses (Figure 6). Together, these observations highlight the structural and regulatory distinctiveness of subfamily II members. Further functional studies—including tissue-specific expression analysis and genetic manipulation in watermelon or related cucurbits—are needed to elucidate the roles of this lineage-restricted subfamily.

Analysis of gene structure and conserved motifs provided further insights into the potential functional divergence among *ClPYL* subfamilies (Figure 4). The variation in exon-intron structure, particularly the presence of single-exon genes in subfamily III, suggests distinct evolutionary paths and possible differences in transcriptional or post-transcriptional regulation compared to multi-exon members in other subfamilies. Consistently, all identified ClPYL proteins contain the canonical START domain (represented by motifs 3 and 5), confirming their fundamental role as ABA receptors [9]. Notably, the distribution of other conserved motifs is closely aligned with subfamily classification. For instance, the unique presence of motifs 4, 5, and 7 in the subfamily II and motifs 8 and 9 in the subfamily I. The observed conservation within and divergence between subfamilies suggests the possibility of functional specialization within the *ClPYL* family, although this hypothesis awaits confirmation through targeted functional assays.

The interplay between *cis*-acting elements and the core promoter region plays a critical role in regulating gene expression, serving as an important pathway for biological signal transduction [37]. For instance, *OsPYL8* and *OsPYL9* are specifically expressed in the endosperm, and their promoters contain multiple motifs associated with endosperm-specific expression in rice (*Oryza sativa*) [38]. The transcription factor ABI5 mediates seed germination in *Arabidopsis* by directly binding to the promoters of *PYL11* and *PYL12* to regulate their expression [39]. The promoter activity of *StPYL16* was significantly enhanced under drought stress in potato (*Solanum tuberosum*) [40]. As a preliminary indication, promoter analysis of *ClPYLs* revealed multiple stress- and hormone-responsive elements, suggesting a potential but yet-to-be-confirmed involvement of the *ClPYL* family in growth, development, and abiotic stress adaptation (Figure 4). Thus, these predictions require experimental validation to determine whether these *cis*-elements are functionally active under specific conditions.

Our expression profiling under drought, salt, and cold stress revealed that *ClPYL* genes exhibit diverse and dynamic transcriptional responses, providing candidate genes for further functional studies aimed at elucidating their potential involvement in watermelon’s abiotic stress signaling networks (Figure 6). The response is often subfamily-associated but stress-specific. For instance, while most members of subfamily III were upregulated under cold stress, they were largely downregulated under drought. *ClPYL05* under drought, *ClPYL02* and *ClPYL08* under salt, and *ClPYL12*, *ClPYL14*, and *ClPYL15* under cold were significantly upregulated at all time points examined, emerging as candidate stress responders potentially involved in long-term adaptive responses. The conserved core function of *PYL* genes in abiotic stress response is further underscored by functional studies in other species. *StPYL8-like* not only robustly responds to abiotic stresses, but also enhances drought resistance in both transiently and stably transformed tobacco plants by upregulating key stress-responsive genes in potato [41]. Grapevine (*Vitis amurensis*) *VaPYL4* promotes plant growth and development under conditions of cold, salt, and drought stress [42]. Overexpression of common vetch (*Vicia sativa*) *VsPYL5* in transgenic *Arabidopsis* enhanced salt tolerance, which was associated with altered Na^+^ and K^+^ levels resulting from the upregulation of genes involved in ion homeostasis [43]. CmPYL7 positively regulates cold tolerance in oriental melon by interacting with CmPP2C24-like within the ABA signaling pathway, modulating antioxidant defense and osmolyte accumulation [44]. *ClPYL* genes exhibit diverse and stress-specific transcriptional responses to drought, salt, and cold stresses at the gene expression level, which requires further investigation through genetic and biochemical approaches.

## 5. Conclusions

This study established a comprehensive genomic framework for the *PYL* gene family in watermelon, systematically characterizing 15 *ClPYL* members in terms of their chromosomal distribution, evolutionary relationships, gene structures, conserved motifs, promoter cis-elements, and expression profiles under drought, salt, and cold stresses. Key findings include the identification of four subfamilies, with subfamily II being uniquely present in cucurbits; subfamily-specific patterns in exon-intron organization and motif composition; distinct distributions of stress- and hormone-responsive cis-elements across subfamilies; and diverse, stress-specific transcriptional responses, with *ClPYL02*, *ClPYL05*, and *ClPYL15* emerging as candidate stress-responsive genes. Collectively, these results provide a foundation for functional studies. Future work will focus on validating the roles of candidate genes—particularly the cucurbit-specific subfamily II—through genetic transformation, protein interaction assays, and phenotypic analyses under stress conditions, thereby enabling the development of stress-tolerant watermelon varieties.

## Figures and Tables

**Table 1:** qRT-PCR primers.

Primer Name	Primer Sequence (5′-3′)
qRTPCR-ClACT-F	GTCGTACAACAGGTATTGTG
qRTPCR-ClACT-R	AAGGTCCAGACGGAGGATAG
qRTPCR-ClPYL1-F	TTGTGGTGGATGTGCCTGAAGGA
qRTPCR-ClPYL1-R	GCACTGCAAGCCGCTCTGAA
qRTPCR-ClPYL2-F	CGAATTACCGCTCCACCACCAC
qRTPCR-ClPYL2-R	TGTACGAGCCAGCCACTTGAGA
qRTPCR-ClPYL3-F	TGCTCCGCCGTCGTTCAAGA
qRTPCR-ClPYL3-R	TCTCAAGCCGCTCGGTACTGTT
qRTPCR-ClPYL4-F	AGGCATCACCGTCACCATCC
qRTPCR-ClPYL4-R	TGTGATCTCCACCGACGATTCT
qRTPCR-ClPYL5-F	GGCTCCTGTTCCTCTTGTTTGG
qRTPCR-ClPYL5-R	TTCGGTGCTTGTGGTGGCT
qRTPCR-ClPYL6-F	TCGTTCGCAGCTTCGATAATCC
qRTPCR-ClPYL6-R	ACCACCGTCACCTCTCTAATGC
qRTPCR-ClPYL7-F	ACGGGTTGTTTCGGGCTTCA
qRTPCR-ClPYL7-R	CCCACATTGCTTGCTTCCAGTT
qRTPCR-ClPYL8-F	GCCGCCGCAACTGCTATGAA
qRTPCR-ClPYL8-R	ATTGTCGAACCGTCGCACCAC
qRTPCR-ClPYL9-F	CCTCCACCGACACGATGTTGT
qRTPCR-ClPYL9-R	GCCACGAACGACCACACGAT
qRTPCR-ClPYL10-F	AACTCTACGGCGAAGTGGGAAG
qRTPCR-ClPYL10-R	TGGACCAGCTAACGACGGAATC
qRTPCR-ClPYL11-F	CCACAGCCACAATCCCACAGAT
qRTPCR-ClPYL11-R	CCCTCACCACACACCGACTAAC
qRTPCR-ClPYL12-F	CGACCACCACCAGCACGATT
qRTPCR-ClPYL12-R	GCGACGGTACAGCTCTTGATGA
qRTPCR-ClPYL13-F	AGAGCTGTACGGTGAGCGAAGG
qRTPCR-ClPYL13-R	CGCCTCCGATGATGCTGAATCC
qRTPCR-ClPYL14-F	TCCGATTCAGTCACCGCCTCAA
qRTPCR-ClPYL14-R	AGCGTTCCGACGTTGCCATC
qRTPCR-ClPYL15-F	GCCGTGGTATGGTCGTTAGTCC
qRTPCR-ClPYL15-R	CTTCAGCCTGTGGTCTCCTCCT

**Table 2:** Information on *PYL* gene family members in watermelon.

Gene Name	Gene ID	Location (Strand)	Number of Amino Acid	Molecular Weight (Da)	Isoelectric Point
*ClPYL01*	Cla97C01G000570.1	Chr01: 398587–400340 (+)	195	21,852.89	6.44
*ClPYL02*	Cla97C03G058970.1	Chr03: 8287359–8287847 (−)	162	17,696.16	5.67
*ClPYL03*	Cla97C04G073350.1	Chr04: 21016155–21016772 (−)	205	22,338.22	6.44
*ClPYL04*	Cla97C05G081110.1	Chr05: 915813–917114 (+)	185	20,777.76	7.69
*ClPYL05*	Cla97C05G090960.1	Chr05: 8965533–8967752 (−)	184	20,612.50	5.98
*ClPYL06*	Cla97C05G099080.1	Chr05: 28240188–28241114 (−)	188	20,916.53	5.25
*ClPYL07*	Cla97C07G132410.1	Chr07: 4148872–4149372 (+)	166	18,721.43	4.76
*ClPYL08*	Cla97C07G133100.1	Chr07: 5508171–5508872 (−)	233	24,916.91	6.78
*ClPYL09*	Cla97C08G155310.1	Chr08: 23334045–23334536 (−)	163	18,297.82	4.83
*ClPYL10*	Cla97C08G155320.1	Chr08: 23338046–23338540 (−)	164	18,123.41	4.89
*ClPYL11*	Cla97C09G172410.1	Chr09: 8805878–8807296 (−)	181	20,352.17	6.13
*ClPYL12*	Cla97C09G174770.1	Chr09: 12072602–12073294 (−)	230	25,469.50	5.32
*ClPYL13*	Cla97C10G186260.1	Chr10: 1618804–1619772 (−)	224	24,987.69	4.97
*ClPYL14*	Cla97C10G205730.1	Chr10: 34694402–34695022 (+)	206	22,466.36	6.54
*ClPYL15*	Cla97C11G212910.1	Chr11: 6257955–6258572 (+)	205	22,393.45	8.22

## Data Availability

All relevant data can be found within this manuscript.
